# Ion Mobility in Thick and Thin Poly-3,4 Ethylenedioxythiophene Films—From EQCM to Actuation

**DOI:** 10.3390/polym13152448

**Published:** 2021-07-26

**Authors:** Rudolf Kiefer, Daniel Georg Weis, Bharath Kumar Velmurugan, Tarmo Tamm, Gerald Urban

**Affiliations:** 1 Conducting Polymers in Composites and Applications Research Group, Faculty of Applied Sciences, Ton Duc Thang University, Ho Chi Minh City 700000, Vietnam; 2 Institute of Physical Chemistry, Albert-Ludwigs-Universität Freiburg, Albertstraße 21, D-79104 Freiburg im Breisgau, Germany; dgweis@gmx.de; 3 FMF—Freiburger Materialforschungszentrum, University of Freiburg, Stefan-Meier-Straße 21, D-79104 Freiburg im Breisgau, Germany; urban@imtek.de; 4 Department of Medical Laboratory Science and Biotechnology, Asia University, Taichung 401, Taiwan; bharathvel@gmail.com; 5 Intelligent Materials and Systems Lab., Faculty of Science and Technology, University of Tartu, Nooruse 1, 50411 Tartu, Estonia; tarmo.tamm@ut.ee; 6 IMTEK—Institute for Microsystem Technology, Laboratory for Sensors, Georges-Koehler-Alle 103, D-79110 Freiburg im Breisgau, Germany

**Keywords:** polymerization potentials, EQCM, cyclic voltammetry, ion flux

## Abstract

Conductive polymer actuators and sensors rely on controlled ion transport coupled to a potential/charge change. In order to understand and control such devices, it is of paramount importance to understand the factors that determine ion flux at various conditions, including the synthesis potential. In this work, the ion transport in thinner poly-3,4-ethylenedioxythiophene (PEDOT) films during charge/discharge driven by cyclic voltammetry is studied by consideration of the electrochemical quartz crystal microbalance (EQCM) and the results are compared to the actuation responses of thicker films that have been synthesized with the same conditions in the bending and linear expansion modes. The effects of polymerization potentials of 1.0 V, 1.2 V, and 1.5 V are studied to elucidate how polymerization potential contributes to actuation, as well the involvement of the EQCM. In this work, it is revealed that there is a shift from anion-dominated to mixed to cation-dominated activity with increased synthesis potential. Scanning electron microscopy shows a decrease in porosity for the PEDOT structure with increasing synthesis potential. EQCM analysis of processes taking place at various potentials allows the determination of appropriate potential windows for increased control over devices.

## 1. Introduction

For the last decade, a significant amount of research has been dedicated to conductive polymer (CP)-based actuators with bending [1,2] or linear [3,4] actuation modes (including fiber-based materials [5,6]) for applications in micro-actuators [7], biomedical devices [8], smart textiles [6,9], and more. Polypyrrole (PPy) has been a popular choice due to its typically higher strain or displacement output, as well as the possibility for electropolymerization in aqueous solutions, which presents suitability in biomedical [10] and biosensor [11] applications, among others [12]. Poly-3,4-ethylenedioxythiophene polystyrenesulfonate (PEDOT:PSS), also soluble in aqueous solutions has made a comeback with the growing popularity of 3D printing [13] in the field of soft robotics [14]. PEDOT, which is formed by electropolymerization, has not been as intensively studied for actuator materials as PPy, although it is well known for its high electrochemical activity, conductivity, and stability [15].

It may seem strange that while so much work has been dedicated to the development of CP actuators, with a plethora of ideas and prototypes published, real applications are still lacking. The main reason for this appears to be a limited control over materials. With only small changes, such as the solvent [16], electrolyte [17], electrochemical polymerization techniques, or temperature [18], one can obtain CP films with rather different actuation properties, including the given actuation direction. The simplified mechanism of CP response (such as PPy or PEDOT) refers to the Faradaic processes where mobile charges are formed on polymer chains in CP films upon oxidation, which immediately creates a force that causes counterions provided by the electrolyte (with or without a solvent) to approach the chains. The influx of ions and the solvent into the polymer film leads to a change in volume, i.e., undergo expansion. Upon reduction, the charges on the chains are reduced and the counterions (with solvents) leave the polymer film, causing the film to shrink, i.e., undergo contraction. In an ideal case, we have only one mobile ion species that triggers the actuation, which could be either anion-driven or, if immobile anions stayed in the CP, cation-driven. In the latter case, this results in expansion upon reduction (known for PPy/DBS [19], as well as PEDOT/CF_3_SO_3_ [20] and PEDOT:PSS [21]); however, mixed-species actuation is observed in real materials and over wider potential windows, for instance, in the case of PEDOT/PF_6_ films [22,23], which can be observed to experience less intensive expansion upon reduction or oxidation.

In order to move from labs and prototypes to real applications, it is of paramount importance to fundamentally understand the factors governing the mobility of ionic species in CP films and their coupling with a polymer. Since PEDOT has enjoyed significantly less attention, we focus on this material here. The electrochemical quartz crystal microbalance (EQCM) has been shown to be an indispensable property for studying mobile species in CP films [24]; however, due to the entailing viscoelastic effects, an increasing film thickness that is meaningful for use as an actuator in EQCM studies cannot be reliably analyzed [25]. Consequently, only thin films are investigated with EQCM techniques.

Both linear (freestanding) and bending (as bilayers) actuators are considered here with PEDOT deposited under a range of different polymerization potentials. Their behaviors are compared to the EQCM results for thinner films that were prepared under the same conditions. Tetrabutylammonium-hexafluorophosphate (TBAPF_6_) in propylene carbonate (PC) was chosen as the electrolyte here, which is an electrolyte that usually results in a mixed ion activity. To the best of our knowledge, this is the first time a comparative study of the EQCM and actuation behavior has been presented for PEDOT films.

SEM and EDX spectroscopy are used to provide additional information for the PEDOT-FF (PEDOT free standing films) samples and all electrochemical experiments (including electro-synthesis) are carried out in triplicate.

## 2. Material and Methods

### 2.1. Chemicals

Polyethylene terephthalate (PET, thickness of 12 μm) foil was obtained from Ecoplast LLC (Khmelnitskiy, Ukraine) and was used as supplied. AT-cut quartz crystals (5 MHz) of a 15 mm diameter were obtained from KVG Quartz Crystal Technology GmbH (Neckarbischofsheim, Germany). Propylene carbonate (PC, 99%), ethanol (technical grade), 3,4-ethylendioxythiophene (EDOT, 97%), and tetrabutylammonium-hexafluorophosphate (TBAPF_6_, 99.9%) were purchased from Sigma-Aldrich (Taufkirchen, Germany) and were used as supplied.

### 2.2. Electropolymerization

The PEDOT films were potentiostatically polymerized (CH Instruments Inc., electrochemical workstation Model 440C, Austin, TX, USA) at polymerization potentials of 1.0 V, 1.2 V, and 1.5 V in a 0.1 M EDOT solution with a 0.1 M TBAPF_6_-PC electrolyte applied for the actuator devices and EQCM studies. The three-electrode setup consisted of a stainless steel plate (4.5 cm^2^ with one side covered with tape to assure PEDOT deposition on only one side), Ag/AgCl (3 M KCl) as the reference electrode, and a platinum counter electrode (18 cm^2^) positioned opposite to the stainless steel plate. The polymerization was carried out under a nitrogen atmosphere at room temperature (25 °C). The PEDOT freestanding films (PEDOT-FF) were removed from the stainless steel electrode and washed several times with PC to remove excess monomers and electrolytes and was then stored for further use with a 0.1 M TBAPF_6_-PC electrolyte. Different deposition times were applied to compensate for the growth rate of the PEDOT-FF, which depended heavily on the polymerization potential. The polymerization conditions are presented in Table 1.

For the bending actuators of the PEDOT-PET bilayers (PEDOT-BL), the PET was sputtered with 100 nm of platinum on one side (resistivity of 400 Ω/sq) and then cut into a strip that was 1.5 cm in length and 0.5 cm in width. The strip was connected as the working electrode and was immersed in the monomer solution with a free length of 1.2 cm. The Pt-PET strip, as a working electrode, was fixed in a polymerization cell (0.1 M EDOT, 0.1 M TBAPF_6_ in PC at 25 °C under a nitrogen atmosphere) and PEDOT was deposited potentiostatically using a HEKA PG 28 potentiostat/galvanostat (HEKA Electronic GmbH, Reutlingen, Germany). The deposition of PEDOT was monitored and stopped after a charge of 200 mC (~340 mC cm^−2^) was reached for each polymerization. After polymerization, the PEDOT-BL was discharged in the monomer solution at 0.0 V for 10 min. For each PEDOT-BL or PEDOT-FF sample, at least three independent polymerizations were carried out and the values given in Table 1 are mean values with standard deviations. The PEDOT-BL and PEDOT-FF samples were washed several times in PC to remove the monomer and electrolyte and were then dried in an oven at 60 °C (2 mbar) for 12 h.

### 2.3. Actuation Measurements

For PEDOT-FF, an in-house linear actuation measurement device was applied and was controlled by in-house software [26] to connect the change of length with the electrochemical signals in real time. The PEDOT-FF samples were cut into lengths of 1.2 cm with a width of 0.4 cm and were fixed between the force sensor and a lower static clamp (free length between clamps of 4 mm at constant force of 9.8 mN) with gold contacts. The PEDOT-FF was set as the working electrode with an Ag/AgCl (3M KCl) reference electrode and a platinum sheet (18 cm^2^) as a counter electrode in a three-electrode cell. Electrochemical measurements via cyclic voltammetry (scan rate 5 mV s^−1^ at ±1 V) and square wave potential steps (0.017 Hz, 0.8 V to 0 V, for long term cycling) were taken with the use of a 0.1 M TBAPF_6_ –PC electrolyte.

PEDOT-BL samples (as working electrode) were fixed in a three-electrode quadratic cell with a 0.1 M TBAPF_6_-PC with Ag/AgCl (3 M KCl) reference electrode and a platinum sheet as a counter electrode. The electrochemical measurements were controlled by a Jaissle potentiostat (Type 1002 PC.T, IPS Elektroniklabor GmbH & Co. KG, Münster, Germany). The movement of the bilayer over a background of millimeter paper was recorded with a CCD camera (EHD kam06, EHD Imaging GmbH, Damme, Germany) connected to a PC-installed frame grabber card (The Imaging Source DFG/LC1) during the electrochemical measurements (cyclic voltammetry and square potential steps as above). From each recorded movement of the PEDOT-BL, images from the video (captured at 25 frames/s) were extracted and the displacement was read at the tip with a careful selection criterion of measuring samples which did not twist in an out-of-plane direction during the actuation.

### 2.4. EQCM Measurements

The working principle of the electrochemical quartz crystal microbalance (EQCM) [27] is based on an inverse piezoelectric effect with a quartz crystal working at its resonance frequency. The changes in the mass of the deposited conductive polymer can be determined during reversible redox cycles (ions and solvent exchanged) by the frequency change. The cut AT crystal, with a 15 mm diameter (oscillation area 0.28 cm^2^), was coated on both sides with a thin layer (20 nm) of chromium to improve the stability of the following platinum layer that was in the range of 60 nm and was coated via vacuum deposition. The platinum-coated quartz was clamped between two O-rings with one side facing the solution and the other side facing air. An Amel potentiostat (Amel model 533, Milano, Italy) was used to control a three-electrode cell with the platinum-coated quartz as the working electrode, an Ag/AgCl wire reference electrode, and a platinum mesh as a counter electrode. Measurement and data collection was performed with in-house software with a Hameg HM 2122 frequency counter (HAMEG Instruments GmbH, Mainhausen, Germany) connected to an IEEE-488 interface bus (Hewlett-Packard, Palto Alto, CA, USA). The potentiostatic polymerizations (1.0 V, 1.2 V, and 1.5 V) took place in a monomer solution under an argon atmosphere (0.1 M EDOT, 0.1 M TBAPF_6_ in propylene carbonate), until the resonance frequency decreased by 5 kHz, which corresponds to a mass addition of 14 μg (i.e., a film thickness in range of 400–500 nm). After polymerization, the polymeric layer was discharged at 0.0 V in the monomer solution. Cyclic voltammetry of the PEDOT-deposited quartz at different polymerization potentials (scan rate 10 mV s^−1^) was performed in an electrolyte solution (0.1 M TBAPF_6_ in PC) and the changes in frequency (mass) during reversible redox cycles with a potential range of ±1.0 V were recorded. The correlation of frequency change (∆*f*) to mass change (∆*m*) in an electrochemical process, with a good linear approximation with a constant *C_QMW_* value (gravimetrical proportionality constant), is given in by the Sauerbrey equation (Equation (1)) [28].

(1) $$\Delta m=C_{QMW}\cdot \Delta f$$

The relationship between the changes of mass on the electroactive surface (∆*m^el^*, g) to changes of charge (∆*Q*, C) is shown in Equation (2) as the modified faradaic law.

(2) $$\Delta Q=n\cdot z\cdot F=z\cdot F\cdot \frac{\Delta m^{el}}{M_{R}}$$

where *F* is the Faraday constant (96,492 C mol^−1^), n is the mole number, z is the number for electrons, and *M_R_* (g mol^−1^) is the molar mass change in the reaction. It needs to be considered that the active surface (A) between the conductive polymer (PEDOT) *A_el_* and the AT quartz crystal (*A_osc_* with mass change ∆*m*_osc_) can be different. As such, Equation (3) denotes the densities of those surfaces (ρ = ∆*m_el_*/*A_el_* = ∆*m_osc_*/*A_osc_*), leading to linear dependence between the change of frequency ∆*f* to the change of charge ∆*Q*:

(3) $$\Delta f=\frac{M}{z\cdot F\cdot C_{QMW}}\cdot \frac{A_{osc}}{A_{el}}\cdot \Delta Q=\frac{M}{z}\cdot C_{EQMW}\cdot \Delta Q$$

The electrogravimetric proportionality constant *C_EQMW_* [29] is a product from the gravimetric proportionality constant C_QMW_, the Faraday constant, and the quotient of surfaces *A_osc_*/*A_el_*. The equation relates to univalent anions/cations, and, as such, the value for “*z*” is 1. The term C_EQCM_ is influenced by the experimental setup and must be calibrated (i.e., measured) using metal deposition for a known thickness of Cr and Pt, as well as the area of the electrically oscillated surface (*A_el_* = 30.8 mm^2^, *A_osc_* = 27.38 mm^2^), the density of the quartz (ρ_Q_ = 2.65 10^6^ g m^−3^), the speed of the shear wave (v_Q_ = 3340 m s^−1^), and the resonance frequency (f_Q_ = 5 MHz), which, in our case, led to a value for *C_EQMW_* of −1.98 mol Hz g^−1^mC^−1^ and −4.85 ng Hz^−1^ for the gravimetric proportionality constant *C_QMW_*. The change of frequency ∆*f* against charge ∆*Q* led to a specific curve where several positions of the slope could be determined, where division by *C_EQMW_* yields the molecular weight of the charge compensating species *M_CCS_* (Equation (4)).

(4) $$\frac{\Delta f}{\Delta Q}=\frac{slope}{C_{EQMW}}=M_{CCS}$$

### 2.5. Characterization

Scanning electron microscopy (SEM) and energy dispersive X-ray spectroscopy (EDX) (Philips XL30-FEG, Philips Electron Optics, Hillsboro, OR, USA) of the PEDOT-BL surfaces at 5 kV were used for analysis after polymerization with the samples that were washed and dried. To obtain better images in the SEM measurement, the samples were sputtered with a thin layer of gold in the range of 20 nm. EDX spectroscopy of the PEDOT-BL samples was carried out at the end of the actuation cycles in an oxidized state at 1.0 V for 5 min and in a reduced state at −1.0 V for 5 min. The surface conductivity values of PEDOT-FF and PEDOT-BL were measured using a 4-point probe conductivity meter (Jandle 4-Point Probe Head, Model RM2, Leighton Buzzard, UK).

## 3. Results and Discussions

The polymerization potential of any conductive polymer is a key factor that influences the properties of the obtained material, including the conductivity, redox activity, ion exchange, and overall actuation. This is illustrated by the linear and bending PEDOT actuators in this work, as the deposition potential is known to influence the PEDOT ion exchange and actuator behavior [23], potentially even playing a role in the actuation direction. In order to make a distinction between the ionic species and the solvent, EQCM measurements were gathered for PEDOT deposited under the same conditions but with a thinner film.

### 3.1. SEM and EDX Spectroscopy of PEDOT Samples

**Figure 1:**
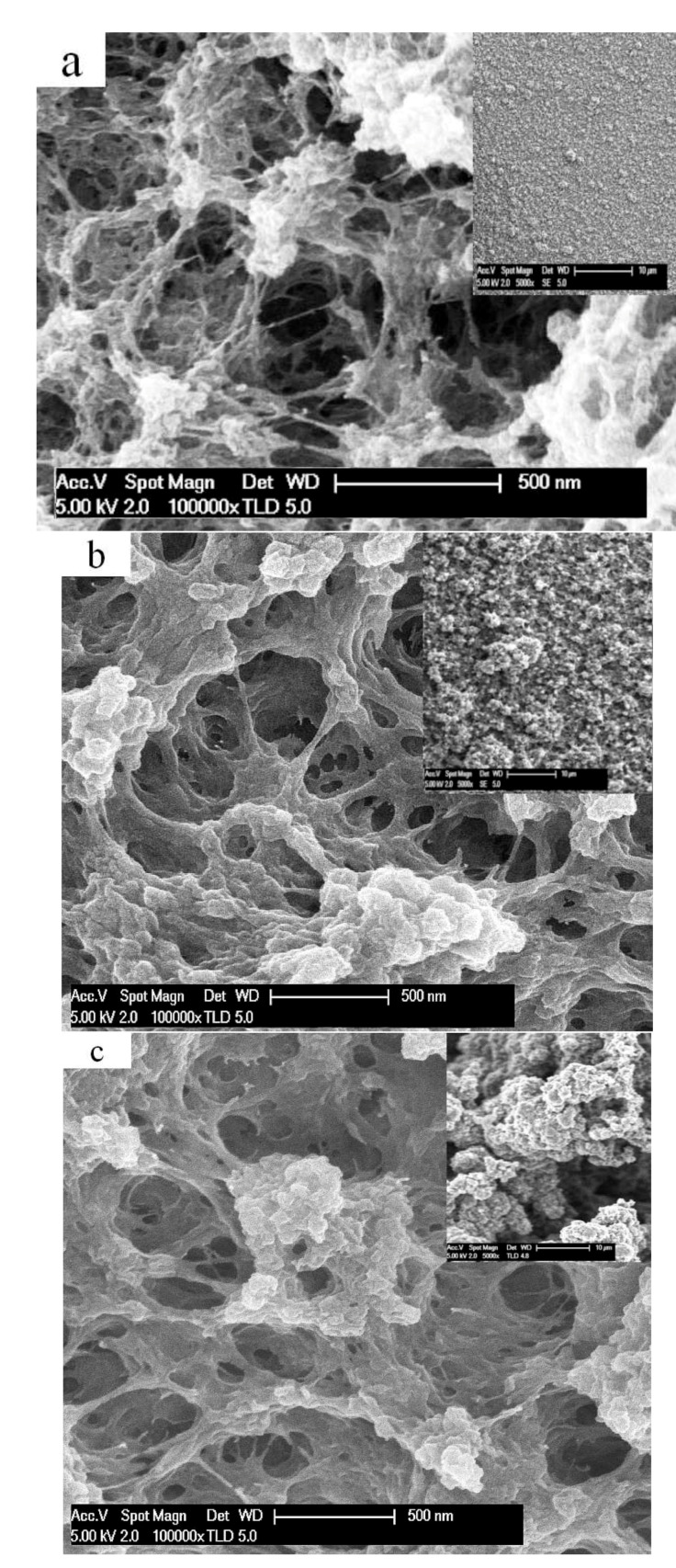
SEM surface images (scale bar denotes 500 nm) with insets (scale bar denotes 10 μm) of PEDOT-BL deposited at different polymerization potentials, E_P_, in a TBAPF_6_-PC electrolyte. (**a**) E_P_ of 1.0 V; (**b**) E_P_ of 1.2 V; (**c**) E_P_ of 1.5 V.

Figure 1a–c present SEM surface images of the PEDOT-BL samples deposited at different polymerization potentials.

All the films showed surfaces that were rather open and highly porous, while those for the lowest polymerization potential of 1.0 V had the most structured and porous appearances and became somewhat more fused and compacted with a higher polymerization potential; however, there were many more distinct differences for the general smoothness, which was significantly higher for the E_p_ 1.0 V material, where the surface became rougher with an increasing synthesis potential. This has been demonstrated before in [23,30]. With a higher polymerization potential, the driving force for the reaction is increased, which means the polymerization time is shorter (Table 1). As a result, PEDOT deposition at a higher potential is more greatly controlled by kinetic factors, leading to less ordered polymer films, and the films also become more brittle with a higher polymerization potential. This likely occurs due to increased crosslinking and other bond formation errors. In general, PEDOT is well known for being less sensitive to overoxidation in comparison to polypyrrole due to the substitution of β-carbons. The conductivity results for PEDOT-BL and PEDOT-FF at different polymerization potentials, E_P_, are presented in Table 2.

While it is difficult to directly compare the conductivities of PEDOT-FF to PEDOT-BL due to the different thicknesses, as well as the presence of the Pt layer on the back side of the bilayer, it can be clearly seen that conductivity decreases with an increased deposition potential for both materials. Moreover, the differences are steeper for 1.0 V to 1.2 V than from 1.2 to 1.5 V. As seen above in the SEM micrographs, a sharper difference in the structure was found as a result, as is also the case for the former potential range. Apparently, the transition from thermodynamic control to kinetic control of the formation process takes place primarily in that potential range, at least under the conditions chosen here.

**Figure 2:**
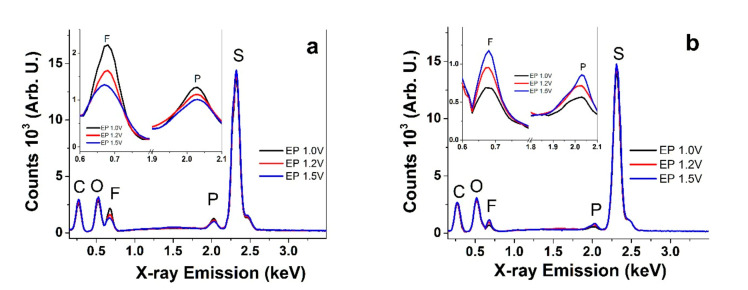
EDX spectra after actuation cycles for PEDOT-BL polymerized at different potentials where E_P_ = 1.0 V (black), E_P_ = 1.2 V (red), and E_P_ = 1.5 V (blue). The insets denote the enhanced peaks of fluorine and phosphorous. (**a**) Oxidized films (polarized 5 min at 1.0 V). (**b**) Reduced films (−1.0 V, 5 min).

To determine which ions accompanied the redox switching, EDX spectroscopy was carried out in oxidized and reduced states, and the results are presented in Figure 2a,b, respectively.

The typical peaks for PEDOT films are found at 0.26 keV for carbon (C), 0.52 keV for oxygen (O), 0.67 keV for fluorine (F), 2.04 keV for phosphorous (P), and a strong peak for sulfur (S) at 2.32 keV. The relatively strong peaks of sulfur (PEDOT ring) and oxygen (the dioxy bridge in PEDOT) did not change during oxidation or reduction (Figure 2a,b). The fluorine and phosphorous peaks refer to the anion PF_6_^-^ from TBAPF_6_. The nitrogen peak from the cation TBA^+^, found in general at 0.38 keV, was not resolved in the spectra, instead being fused with the strong carbon and oxygen peaks. The insets in Figure 2a,b emphasize the changes in the fluorine and phosphorous peaks, revealing that the fluorine and phosphorous peaks were strong upon oxidation and decreased with an increased polymerization potential. The fluorine peak decreased significantly upon reduction; however, the order was now reversed, with the peak intensity increasing with the synthesis potential. Overall, the spectra indicate that some of the anions are trapped in the films, with immobilization increasing with an increasing deposition potential. Cations must balance the charge in such cases, thereby resulting in mixed-mode ion transport and actuation.

### 3.2. Cyclic Voltammetry Driven Ion Exchange

Cyclic voltammetric measurements in the voltage range of ±1.0 V for PEDOT-FF and PEDOT-BL deposited at different polymerization potentials were gathered to investigate the response behaviors with a TBAPF_6_-PC electrolyte. Cyclic voltammetry is the optimal method to analyze redox cycles, whereas the CV shapes and the concurrent actuation response can help to identify oxidation/reduction processes and ion flux caused by a change in volume. By avoiding overoxidation and overreduction, the charging/discharging coulovoltammetric responses showed closed loops, corresponding to a steady state condition [31] (charging/discharging in balance) (Figure S1a,b).

#### 3.2.1. PEDOT Linear and Bending Actuation

**Figure 3:**
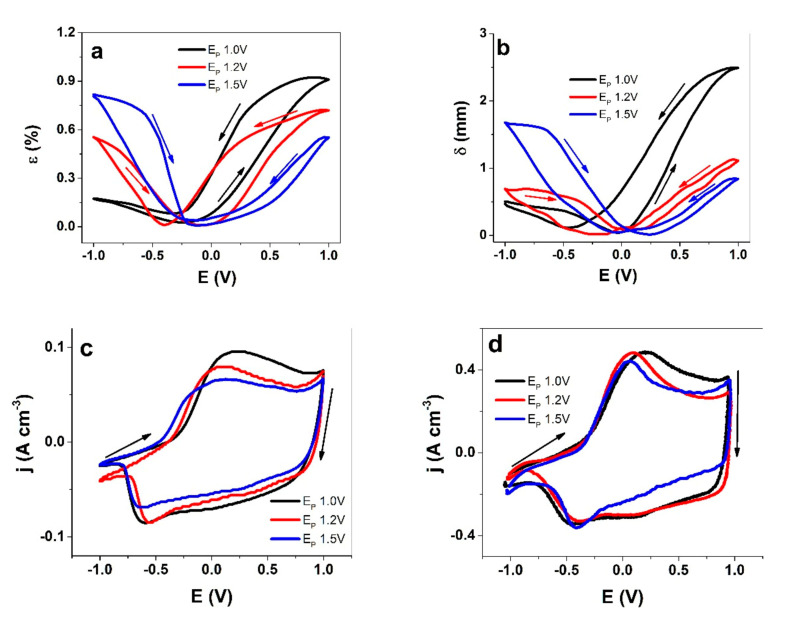
Actuation and current density responses of cyclic voltammetry (scan rate of 5 mV s^−1^, 3rd cycle) for PEDOT samples polymerized at E_P_ = 1.0 V (black line), E_P_ = 1.2 V (red line), and E_P_ = 1.5 V (blue line). (**a**) Strain (ε) for PEDOT-FF; (**b**) bending displacement (δ) of PEDOT-BL; (**c**) current density (j) of PEDOT-FF; (**d**) current density (j) of PEDOT-BL against the potential (E, ±1.0 V) in the electrolyte TBAPF_6_-PC. The arrows indicate the scan direction (starting point of −1.0 V).

While PEDOT was less sensitive towards the synthesis temperature than, for instance, polypyrrole, the PEDOT-FF polymerized at room temperature was stiff and brittle, which limits linear actuation in comparison to other samples prepared at low temperatures. The curves of the cyclic voltammetry (scan rate of 5 mV s^−1^) for driven linear actuation, as well the bending displacement of PEDOT-BL, are presented in Figure 3a,b, respectively. The current density curves of the PEDOT-FF films are shown in Figure 3c and those of PEDOT-BL are presented in Figure 3d.

All the strain curves of PEDOT-FF (Figure 3a), as well as the bending displacement curves of PEDOT-BL (Figure 3b), which were deposited at different polymerization potentials, showed mixed actuation modes of expansion upon both oxidation and reduction; however, there was a clear tendency that with an increasing polymerization potential, the expansion upon oxidation decreased and the expansion upon reduction increased, corresponding to a shift from anion-dominated to cation-dominated activity. Taking into consideration that an increasing polymerization potential changes the morphology and compactness of PEDOT, the denser, less porous (Figure 1c), and more cross-linked PEDOT networks created at higher polymerization potentials would partly hinder the flux of the incorporated (solvated) PF_6_^-^ anions, thus limiting the rate and extent of the charge the anions can compensate for. Hence, the expansion upon reduction is a consequence of TBA^+^ cation ingress to maintain electroneutrality. The trapped PF_6_^-^ anions remaining inside the polymer matrix during reduction can also lead to an increase in the osmotic pressure [32], influencing the solvent content, and thus the swelling during reduction. Another theory by Hillman et al. [33] states that solvent uptake during doping and de-doping depends on the solvent properties. The PC used here might be transported more easily in PEDOT due to its hydrophobic nature. In either case, the differences in the polymerization potentials clearly resulted in structure changes, which is the main factor for increased expansion upon reduction.

The current density of PEDOT-FF (Figure 3c) was much lower than that reached by PEDOT-BL (Figure 3d), which can be primarily explained with the 50% lower conductivity of the freestanding film when compared to the bilayer. In general, the curves share several common features and similar trends can be observed. The main oxidation peak, which is perhaps most clearly seen for the PEDOT-BL (Figure 3d), shifted from 0.21 V to 0.1 V to 0.07 V for films with E_P_ values of 1.0 V, 1.2 V, and 1.5 V, respectively. This is consistent with the characteristic of increased cation activity. As peaks shifted towards cathodic potentials with an increasing PEDOT deposition potential, this also reduced in intensity, especially on the anodic side of the cycle. A very similar trend can be seen for the PEDOT-FF. The decreasing cycling current density with an increasing formation potential can be attributed to the decrease in conductivity (Table 2), but also to lower counterion mobility.

All of the charge density versus potential curves in Figure S1a,b represent closed cycles, indicating that charging/discharging was in balance for this potential range (±1 V) [31]. Table 3 compares the strain and bending displacement upon oxidation and reduction, as well as the charge densities of PEDOT-FF and PEDOT-BL.

The comparison between the responses of PEDOT-FF and PEDOT-BL shown in Table 3 underline the trends discussed above, i.e., with a higher deposition potential, the strain and bending displacement on the reduction side increases at the expense of the oxidation side. If the net displacement is considered, which is calculated from the differences between the displacements for oxidation and reduction, the mixed ion activity actuators are not ideal options. The typical design goal is to avoid such cases, i.e., trying to obtain approximately pure ion species activity with a single actuation direction for expansion upon either oxidation or reduction. One possible approach to suppress the activity of one ionic species is to embed ion-selective additives, like polymerizable ionic liquids [34] into the CP matrix, which, by maintaining the positive charge independent of the conducting polymer redox state in the blend, leads to selectively anion-active materials, thus primarily resulting in expansion upon oxidation. Without a dedicated selective system, the mobile species can even change over time, as shown recently using electrochemistry and AFM, where, with increased cycling, PEDOT films with a LiClO_4_ aqueous electrolyte changed their mode from anion-driven to cation-driven [35]. As such, by either selecting different electrolytes or solvents or choosing the right polymerization potential, one can promote the desired control of actuation by avoiding mixed modes. On the other hand, mixed actuation with virtually equal strain upon oxidation and reduction allows mirrored and linear trilayer actuators to be constructed [36]. In either case, it is of paramount importance to understand the factors influencing the actuation mode, which in turn depends on the balance of the mobile species. A relatively small spherical anion like PF_6_^-^ is expected to move in and out of a polymer matrix more easily than bulkier anions, but the actuation results shown here demonstrate otherwise. Consequently, EQCM measurements for thinner films were gathered at the same polymerization potential.

#### 3.2.2. EQCM Measurements

**Figure 4:**
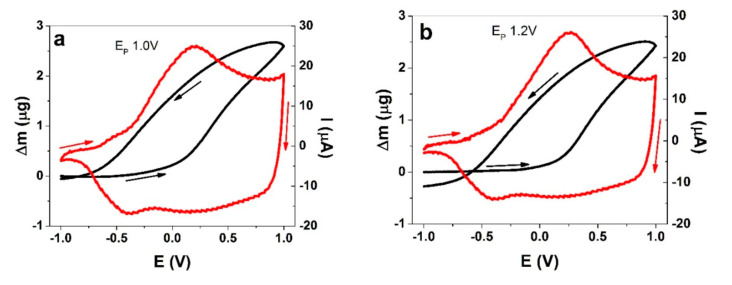

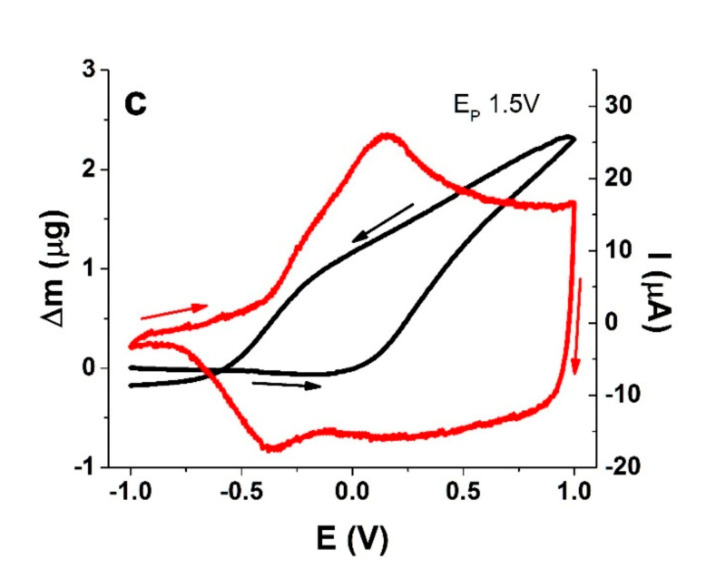
Cyclic voltammetry-driven (±1.0 V, scan rate of 10 mV s^−1^, current denoted by the red curve) EQCM measurements (mass change denoted by the black curve) for a TBAPF_6_-PC electrolyte with PEDOT films polymerized at different polymerization potentials: (**a**) E_P_ = 1.0 V; (**b**) E_P_ = 1.2 V; (**c**) E_P_ = 1.5 V. The arrows indicate the direction of the scan (starting point of −1.0 V).

While the EQCM can more reliable distinguish between mass changes from viscoelastic effects at lower film thicknesses, it is also known that very thin films have different ion transport properties. Hence, it is important to find a compromise. PEDOT was deposited potentiostatically here at different polymerization potentials that were equal to those used in the aforementioned actuation studies (E_P_ values of 1.0 V, 1.2 V, and 1.5 V) for actuator films. The mass changes accompanying the CV-driven processes for the PEDOT films of different formation potentials are presented in Figure 4. Charge–potential curves are presented in Figure S1c, again revealing that the charging/discharging behaviors were in balance.

EQCM analysis allows one to distinguish between mass balance changes in the various regions of a redox cycle. Upon oxidation from −1.0 V to 0.0 V, all the PEDOT films here presented a slightly negative slope, which was virtually invisible for E_p_ 1.0 V, and the slopes became steeper for higher polymerization potentials. This means that the PEDOT films lost weight in this potential range, as observed before by Kvarnström et al. [37] in a solution of TBAPF_6_ in acetonitrile. As expected, the bending displacement of PEDOT-BL in the same potential range (Figure 3b) showed contraction. In particular, in the case of the films where the E_P_ was 1.0 V, the slight mass loss in the beginning of the oxidative scan may (in addition to cation involvement) also be explained by the current still remaining negative at these potentials (Figure 3c), corresponding to ongoing reduction, even as the oxidative scan had started. The next phase started for the potentials corresponding to the oxidation peak in the CV and progressed all the way to 1.0 V, thereby resulting in a significant mass increase for all PEDOT films (Figure 4a). This meant that ions and solvent molecules had moved in, as demonstrated above by the expansion of the PEDOT-BL (Figure 3b). In the reverse scan, from 1.0 V until the reduction peak at −0.4 V, a loss of mass was recorded (negative slope) for all materials, which correlates with the contraction of PEDOT-BL as shown in Figure 3b. In the case of the PEDOT films where E_P_ = 1.5 V (Figure 4c), a breaking point can clearly be observed at around −0.2 V, which is where the slope changed, indicating a change in the anion-cation participation ratio.

Plotting the frequency change ∆*f* against the charge ∆*Q*, as in Equations (2) and (3), the slopes of the curves (Figure S2a–c) of the different regions give the molecular weights of the compensating charge species, i.e., *M_CCS_* (Equation (4), dimensionless). The obtained *M_CCS_* values indicate the amounts and kinds of ions with or without a solvent (molar mass of PC of 102.09 g∙mol^−1^) that were incorporated or expelled during voltammetric cycling. For TBAPF_6_, the molecular weight of the anion PF_6_^-^ was 144.96 g∙mol^−1^, and that of the cation TBA^+^ was 242.47 g∙mol^−1^. In a case where *M_CCS_* is greater than the molecular weight of the anion or cation, solvent molecules are likely transported, while negative values smaller than the anion or cation molecular weights hint to mixed processes of simultaneous anion and cation exchange, potentially even in combination with solvent molecules. With the EQCM methodology, the exact determination for which ions move in or out in a mixed activity scenario can only be estimated.

**Figure 5:**
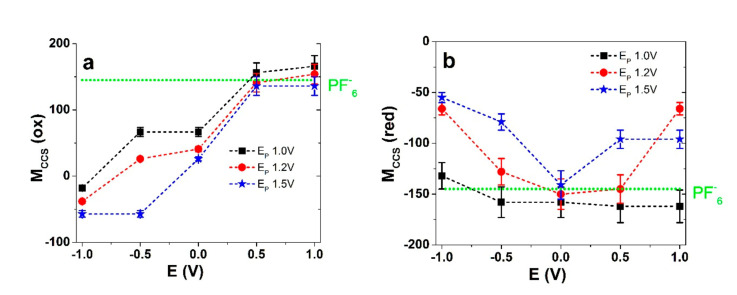
PEDOT deposited at different polymerization potentials where E_P_ = 1.0 V (··■··), E_P_ = 1.0 V (··●··), and E_P_ = 1.5 V (··★··), showing charge compensation for species *M_CCS_* in (**a**) oxidation against the potential direction for −1.0 V to +1.0 V and (**b**) reduction against the potential direction of 1.0 V to −1.0 V. The included dotted green line represents the molar mass for PF_6_^-^ anions.

Figure 5a,b show the values of *M_CCS_* as a function of the cycle potential for the PEDOT samples polymerized at different potentials, with the values separated for oxidation and reduction. For each polymerization potential, at least three independent measurements were taken and the results represent mean values with the error bars of the calculated *M_CCS_* values.

It is immediately clear that the trends for the three polymerization potentials were all different. Upon oxidation where E_P_ = 1.0 V, the *M_CCS_* values (Figure 5a) for the potentials starting from −1.0 V were negative, i.e., −18 ± 2, followed by −38 ± 4 for E_P_ = 1.2 V, and lower still for E_P_ = 1.5 V with −57 ± 6. The following slopes at a potential of −0.5 V revealed positive values for E_P_ = 1.0 V (67 ± 7) and E_P_ = 1.2 V (26 ± 3), while the values were still negative when E_P_ = 1.5 V. The *M_CCS_* values were nowhere near the molecular weights of the ions, where mixed ion actuation takes place from −1.0 V to −0.5 V where cations are expelled and anions are incorporated. This process is likely accompanied by solvent molecules. Vandesteeg et al. [22] likewise proposed cation expulsion instead of anion insertion during oxidation in EDOT-based polymers, but without further investigations. At a potential of 0.0 V the *M_CCS_* values were all positive (but still under the molecular weight of PF_6_^-^ anions corresponding to mixed activity). With increasing polymerization potential, the *M_CCS_* values decreased (higher cation involvement). This corresponds to the switching region in Figure 3a,b, where contraction changed to expansion. Upon further oxidation, (slopes at 0.5 V and 1.0 V), the films where E_P_ = 1.0 V and E_P_ = 1.2 V had *M_CCS_* values in a range above the molecular weight of PF_6_^-^, corresponding to dominant anion incorporation, along with solvent molecules (as seen in Figure 3a,b with the primary expansion upon oxidation). In the case where E_P_ = 1.5 V, the *M_CCS_* value of 136 ± 14 is still slightly lower than the molecular weight of PF_6_^-^, where the dominant process was still anion incorporation but with some mixed characteristics. It has been proposed that the hysteresis of the cycle might be caused by a variable number of trapped ions in PEDOT network in the considered timeframe [38].

As usual, a reduction scan can be more informative when starting from a fully oxidized and conductive state. For the reverse scan (Figure 5b), in the case where E_P_ = 1.0 V, the *M_CCS_* values of −166 and −152 in a potential range from 1.0 V to −0.5 V show a clear indication of (solvated) anion expulsion, thus corresponding to contraction in PEDOT-FF and PEDOT-BL (Figure 2a,b). In the case where E_P_ = 1.2 V, the picture is different. The reduction scan *M_CCS_* values do not reflect those for the oxidation scan. While it might be expected that the same solvated PF_6_^-^ anions left the film, the *M_CCS_* values of −66 ± 6 do not describe that. Similarly, that discussed above can be explained (in addition to the unlikely cation involvement) by the fact that in the beginning of the reduction scan, the potential decrease did not immediately stop incomplete oxidation, and some anions still entered the film before beginning to leave. A further reduction from 0.5 V to 0.0 V showed a *M_CCS_* result of −150 ± 15, which already corresponds to anions with solvent molecules leaving the PEDOT film. By reaching a potential of −1.0 V, PEDOT-BL and PEDOT-FF (Figure 3a,b) showed small expansion, and the mixed ion involvement was reflected here by a *M_CCS_* result of −66 ± 6, as anions were mainly leaving and cations were already moving in (with solvent molecules involved in both processes). The polymerization potential of E_P_ = 1.5 V differed the most, where, during the whole reduction scan, the *M_CCS_* values never reached the anion molecular weight value, corresponding to a mixed ion process throughout, with cation incorporation dominating as the potential dropped, corresponding to a higher strain/displacement for PEDOT-FF and PEDOT-BL upon reduction. The polymerization potentials chosen here have demonstrated significant effects regarding the dominant actuation direction, along with the accompanying ion flux.

To our knowledge, this is the first attempt to analyze the effect of polymerization potential in regard to the anion flux behind the linear and bending actuation modes of PEDOT actuators using EQCM measurements, especially in terms of addressing potential regions individually, thus allowing the tuning of the actuator response by selecting potential windows for desired outcomes. In summary, higher formation potentials of 1.2 V and 1.5 V brought about increased cation involvement in redox processes, while solvent uptake only played a minor role, as discussed before [24]. While some earlier works have shown significant solvent effects [33], their omission of any possible cation involvement does not allow a clear comparison. As seen from the structural description, with an increasing deposition potential, the polymer matrix becomes denser and less permeable for ions. As such, solvation shells are unlikely to remain intact as ions enter the material.

## 4. Conclusions

Charging/discharging in PEDOT or any other conducting polymer is accompanied by ion flux in order to maintain a neutral net charge. PEDOT polymerized different potentials has been studied here in order to establish the effect of the polymerization potential on the ion mobility and the corresponding expansion. Depending on the given design, such findings are potentially applicable for use in linear (PEDOT-FF) or bending (PEDOT-BL) actuators. The mostly anion-dominated activity seen for PEDOT deposited at 1.0 V gradually shifted to increased cation involvement with increasing deposition potentials of 1.2 V and 1.5 V, which in turn led to the a shift from expansion upon oxidation to mixed and increasing expansion upon reduction. EDX spectroscopy confirmed that, upon reduction, an increasing PF_6_- anion content remained in PEDOT polymerized at higher potentials, calling for cation incorporation in order to maintain a neutral net charge. The SEM micrographs indicated that denser and less porous PEDOT networks were formed at higher polymerization potentials, which might be the main reason for lowered PF_6_^-^ mobility. EQCM measurements confirmed that mixed ion activity (both cation and anions participation) processes accompanied charging/discharging. As mixed activity modes are undesirable in general, the extensive study of PEDOT here enables one to understand the factors behind ion mobility and to either choose polymerization potentials optimal for more controlled actuation or to make maximum use of the mixed activity by choosing conditions for equal expansion upon reduction and oxidation. Future applications of such devices, i.e., where one ion movement process dominates, include soft robotics and smart textiles.

## Figures and Tables

**Table 1:** Polymerization parameters of PEDOT-BL and PEDOT-FF with the polymerization time t_P_, charge density Q, current density j (by the effect of polymerization potentials), and thickness d.

E_P_ (V)	t_p_ (min)		Q (mC cm^−2^)		j (mA cm^−2^)		d (μm)	
	BL	FF	BL	FF	BL	FF	BL	FF
1.0	228 ± 14	327 ±22	333 ± 22	356 ± 21	0.05 ± 0.003	0.06 ± 0.004	4.3 ± 0.4	33 ± 3
1.2	5.2 ± 0.3	8.8 ± 0.4	365 ± 22	378 ± 27	1.46 ± 0.1	1.6 ± 0.1	4.8 ± 0.5	34 ± 2
1.5	0.9 ± 0.1	1.2 ± 0.1	367 ± 30	386 ± 32	6.73 ± 0.4	7.8 ± 0.5	5.2 ± 0.4	38 ± 3

**Table 2:** Surface conductivities of PEDOT-BL and PEDOT-FF when polymerized at different potentials in an oxidized state.

E_P_	PEDOT-FF (S cm^−1^)	PEDOT-BL (S cm^−1^)
1.0 V	6.4 ± 0.6	15.8 ± 1.2
1.2 V	3.5 ± 0.3	8.8 ± 0.7
1.5 V	2.5 ± 0.2	5.9 ± 0.5

**Table 3:** Strain ε, bending displacement δ upon oxidation (+1 V)/reduction (−1 V), and charge density Q as per cyclic voltammetry for PEDOT-FF and PEDOT-BL deposited at different polymerization potentials (E_P_). Values are those extracted from three independent experiments and are shown as mean values with standard deviations.

E_P_	PEDOT-FF			PEDOT-BL		
	ε (%) +1 V	ε (%) −1 V	Q (C cm^−3^)	δ (mm) +1 V	δ (mm) −1 V	Q (C cm^−3^)
1.0 V	0.92 ± 0.08	0.16 ± 0.01	98 ± 8	2.5 ± 0.2	0.50 ± 0.03	91 ± 8
1.2 V	0.73 ± 0.06	0.56 ± 0.04	87 ± 8	1.1 ± 0.1	0.70 ± 0.05	85 ± 7
1.5 V	0.55 ± 0.05	0.82 ± 0.07	72 ± 7	0.80 ± 0.06	1.7 ± 0.1	74 ± 7

## Data Availability

The data presented in this study are available on request from the Corresponding author.

## Supplementary Materials

The following are available online at https://www.mdpi.com/article/10.3390/polym13152448/s1: Figure S1. Charge potential curves in cyclic voltammetry (± 1.0 V, TBAPF_6_-PC electrolyte) of PEDOT films made at E_P_ 1.0 V (black curve), E_P_ 1.2 V (red curve) and E_P_ 1.5 V (blue curve) shown in a: for PEDOT-FF, in b: for PEDOT-BL and c: for EQCM measurements. The arrows indicate the start and end points of the 3rd cycle. Figure S2. Cyclic voltammetric (scan rate 10 mV s^−1^) in TBAPF_6_-PC electrolyte at ±1.0 V showing EQCM measurements of frequency f against charge Q of PEDOT films on quartz crystals polymerized at a: E_P_ 1.0 V, b: E_P_ 1.2 V and c: E_P_ 1.5 V. The arrows indicate the direction of the san (starting point −1.0 V).
