# Supplementary material for: Ion Mobility in Thick and Thin Poly-3,4 Ethylenedioxythiophene Films—From EQCM to Actuation
Source: Polymers (Basel). 2021 Jul 26;13(15):2448. doi: 10.3390/polym13152448 (PMC8348298; doi:10.3390/polym13152448)
Supplement: Supplementary file 1 [file polymers-13-02448-s001.zip › polymers-1295010-supplementary.pdf]

Supplementary

Ion Mobility in Thick and Thin Poly-3,4-Ethylenedioxythiophene Films—From EQCM to Actuation

Rudolf Kiefer[1,*], Daniel Georg Weis[2,3], Bharath Kumar Velmurugan[4], Tarmo Tamm[5] and Gerald Urban[3,6]

[1]Conducting polymers in composites and applications Research Group, Faculty of Applied Sciences, Ton Duc Thang University, Ho Chi Minh City, Vietnam

[2]Institute of Physical Chemistry, Albert-Ludwigs-Universität Freiburg, Albertstraße 21, D-79104 Freiburg im Breisgau, Germany

[3]FMF - Freiburger Materialforschungszentrum, University of Freiburg, Stefan-Meier-Straße 21, D-79104 Freiburg im Breisgau, Germany

[4]Department of Medical Laboratory Science and Biotechnology, Asia University, Taichung, Taiwan

[5]Intelligent Materials and Systems Lab, Faculty of Science and Technology, University of Tartu, Nooruse 1, 50411 Tartu, Estonia

[6]IMTEK – Institute for Microsystem Technology, Laboratory for Sensors, Georges-Koehler-Alle 103, D-79110 Freiburg im Breisgau, Germany

*Corresponding author. Tel: +886 905605515. E-mail: rudolf.kiefer@tdut.edu.vn (Rudolf Kiefer)

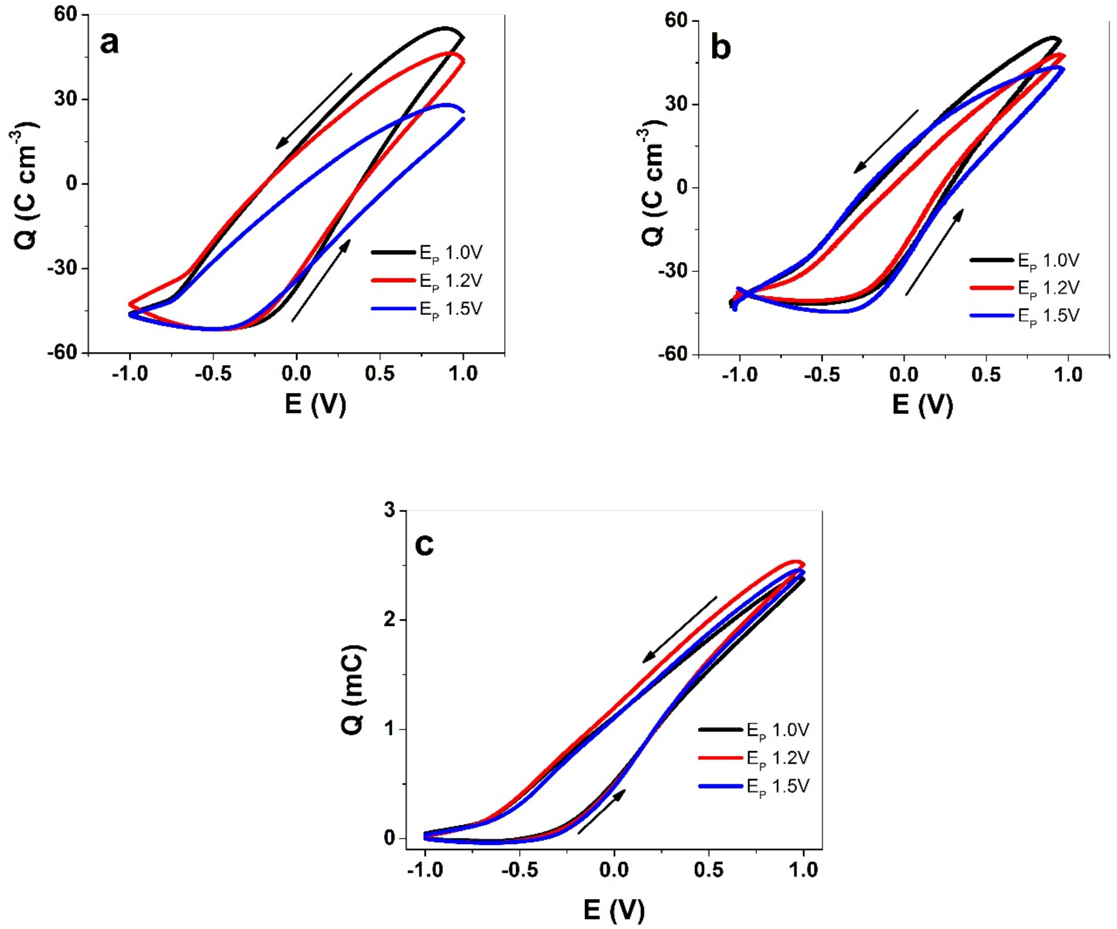

Figure S1. Charge potential curves in cyclic voltammetry (± 1.0V, $TBAPF_6$-PC electrolyte) of PEDOT films made at E_P 1.0V (black curve), E_P 1.2V (red curve) and E_P 1.5V (blue curve) shown in a: for PEDOT-FF, in b: for PEDOT-BL and c: for EQCM measurements. The arrows indicate the start and end points of the 3rd cycle.

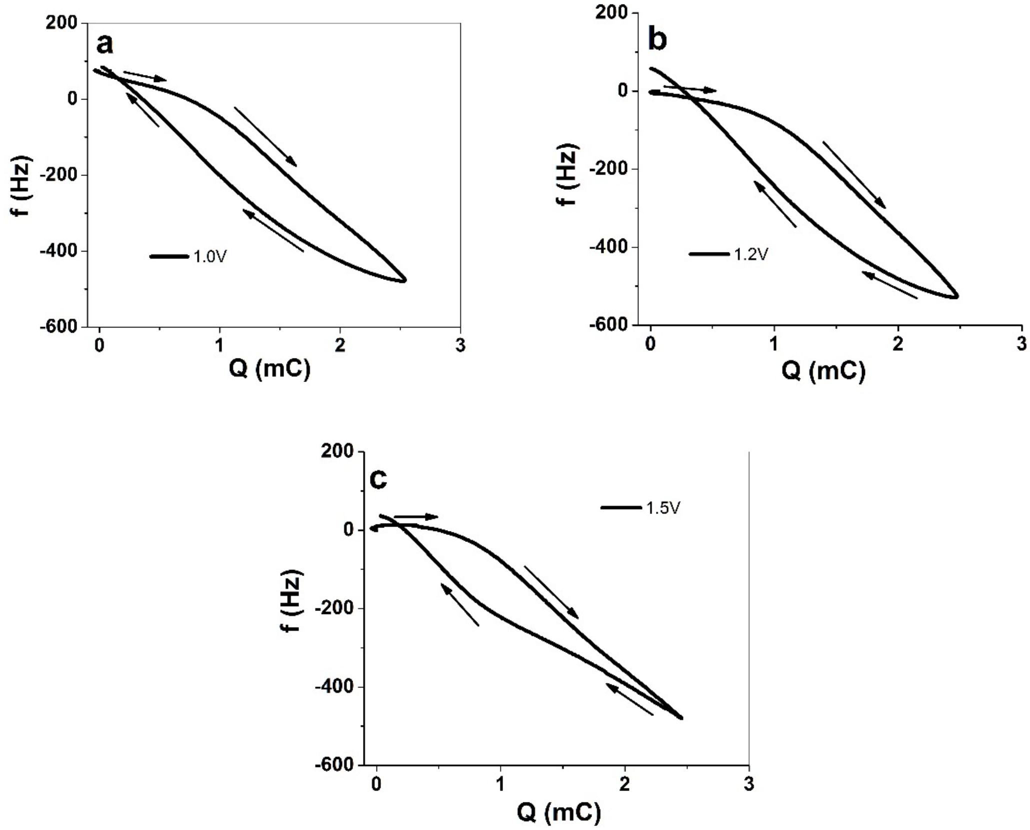

Figure S2. Cyclic voltammetric (scan rate 10 mV s^{-1}) in $TBAPF_6$-PC electrolyte at ±1.0V showing EQCM measurements of frequency f against charge Q of PEDOT films on quartz crystals polymerized at a: E_P 1.0V, b: E_P 1.2V and c: E_P 1.5V. The arrows indicate the direction of the san (starting point -1.0V).
